# A refined reweighing technique for nondiscriminatory classification

**DOI:** 10.1371/journal.pone.0308661

**Published:** 2024-08-20

**Authors:** Yuefeng Liang, Cho-Jui Hsieh, Thomas C. M. Lee

**Affiliations:** 1 Department of Statistics, University of California at Davis, CA, United States of America; 2 Department of Computer Science, University of California at Los Angeles, Los Angeles, CA, United States of America; Xi’an Jiaotong University, CHINA

## Abstract

Discrimination-aware classification methods remedy socioeconomic disparities exacerbated by machine learning systems. In this paper, we propose a novel data pre-processing technique that assigns weights to training instances in order to reduce discrimination without changing any of the inputs or labels. While the existing reweighing approach only looks into sensitive attributes, we refine the weights by utilizing both sensitive and insensitive ones. We formulate our weight assignment as a linear programming problem. The weights can be directly used in any classification model into which they are incorporated. We demonstrate three advantages of our approach on synthetic and benchmark datasets. First, discrimination reduction comes at a small cost in accuracy. Second, our method is more scalable than most other pre-processing methods. Third, the trade-off between fairness and accuracy can be explicitly monitored by model users. Code is available at https://github.com/frnliang/refined_reweighing.

## 1 Introduction

Advances in computing technology enable automated decision-making to be popularized in many social contexts. Artificial intelligence can be more efficient at candidate screening than human resources recruiters [1]. Predictive policing helps forecast crime in law enforcement operations [2]. However, the rising popularity unintentionally introduces socioeconomic and racial disparities [3]. As anti-discrimination laws state that it may be illegal to introduce serious bias [4, 5], discrimination-aware classification has become an important research topic in machine learning [6].

Reweighing (RW) [7] is one of the earliest bias mitigation algorithms studied by researchers. It alleviates sample size disparity [4] by assigning weights to all cohort and label tuples in the training data. Despite its easy implementation [7–12] and accuracy reservation [7], RW has two limitations. First, it ignores potential relation between the within-cohort attributes and other features. If other features are proxies of the within-cohort attributes, the re-weighted data may not be discrimination-free [4]. Second, it does not allow decision makers to control the cost in accuracy they would pay for fairness in real-world problems.

To overcome those two limitations, we propose the Refined Reweighing (RRW) technique that generates more fine-grained weights than RW’s by investigating distributional inequity across *all* attributes in a two-phase process. In Phase I, we calculate the sample sizes of all observed categorical attribute-label combinations and transform them into weights. We formulate the weight assignment as a linear programming problem. If there exist numerical attributes, we proceed with Phase II that integrates their probability distribution with weights obtained in Phase I. The final weight assignment is independent of the ultimate prediction method, which makes RRW versatile.

The empirical results are promising. RRW is competitive with state-of-the-art pre-processing treatments [6, 12, 13] in the AI Fairness 360 toolkit [14] on accuracy and fairness in three classification tasks. As the numbers of data points in those datasets are all under 50, 000, and the numbers of attributes are all below 6, we conduct an extensive simulation study to evaluate its scalability on more attributes and larger sample sizes. For example, RRW manages to handle 40 million instances with 15 attributes in 2 minutes, while it takes some other methods at least 30 minutes to do so. The effectiveness of both optimization phases are also evaluated by simulation studies and real data experiments.

The rest of the paper is organized as follows. We review related work in Section 2 and introduce our method in Section 3. Experimental results are presented and discussed in Section 4, followed by concluding remarks in Section 5.

## 2 Related work

Researchers have studied various fairness measures such as statistical parity [15–17], predictive parity [18] and equalized odds [15, 19]. Indeed, all measures fall into three main categories. *Group fairness* ensures that the subjects in the protected and unprotected cohorts have similar outcomes [13, 20, 21]. *Individual fairness* emphasizes that subjects possessing similar attributes are similarly labeled [16, 22, 23]. *Counterfactual fairness* declares fairness to a subject if the outcome is the same in both the real world and a counterfactual world where that subject belonged to a different cohort based on causal graphs [24]. Our goal is to secure statistical parity, a notion for group fairness.

Discrimination prevention algorithms can mitigate bias at different stages of predictive modeling. *Pre-processing* [6, 10, 12, 13, 25] approaches modify the training data. *In-processing* [26–28] approaches adjust classification models. *Post-processing* [19, 22, 29] approaches change the predicted labels. We focus on pre-processing in this work.

Suppression, resampling, modification and reweighing are four typical techniques in pre-processing approaches [10]. Suppression removes sensitive attributes in training and testing. This removal alone is ineffective when some of the insensitive attributes are highly correlated with the sensitive ones [4]. Resampling refers to stratified sampling applied on all combinations of cohort and labels [10]. Each combination is either under-sampled or over-sampled. Modification changes (1) the attributes, (2) the labels [9], or both [6]. Reweighing assigns weights to all pairs of attributes and label. Data can be freed from discrimination without changing its space or value [10]. The last two techniques are more relevant to RRW. We mainly compare RRW with RW [7] and the three modification algorithms in the AI Fairness 360 toolkit [14] summarized below.

- RW [7] applies appropriate weights to different (cohort, label) tuples in the training data. The weight is equal to the product of the marginal probabilities of cohort and label over the observed probability of their joint distribution. If the data was unbiased, all cohorts and labels would be statistically independent and the weights would be one.
- Learning Fair Representations (LFR) [12] is a prototype-based clustering algorithm. It maps each individual to a probability distribution in a latent representation space in order to obfuscate any cohort-related information, while retaining information of other attributes as much as possible.
- Disparate Impact Remover (DIR) [13] edits attributes from which the protected cohort can be predicted. It explores disparate impact [30, 31], balanced error rate and *ϵ*-fairness to remove the attributes’ ability to distinguish between different cohorts while preserving rank-ordering within cohorts.
- Optimized Pre-Processing strategy (OPT) [6] learns a probabilistic transformation that modifies attributes and labels by taking group fairness, individual distortion and data utility into consideration. An appropriate choice of distortion metric is essential for effective discrimination reduction.

RW and RRW are reweighing methods. LFR, DIR and OPT belong to the modification category mentioned earlier. A fundamental difference between these two groups is that modification alters the *elements* of the instances, while reweighing updates the empirical distribution of the training instances, or their *influence* in a classifier. This distinction leads to their different levels of scalability and training time complexity. RW does not require any optimization, but it does not provide any control for the fairness-accuracy trade-off. Although RRW entails a two-phase optimization, its processing time is much shorter than the amount of time required by most modification methods. DIR requires the user to specify a repair level, a hyper-parameter indicating how much the user wishes for the distributions of the cohorts to overlap. LFR changes the space of the data distribution so that classification predictions are made on prototypes. Hyper-parameters controlling individual fairness, group fairness and prediction accuracy need to be carefully tuned to produce ideal results. OPT involves a large amount of calibration that would potentially undermine its feasibility, and it is only applicable to categorical attributes. The simulation study in Section 4 validates these statements.

## 3 Proposed technique

The main idea of RRW is to attach customized weights to training instances with different sensitive attributes (cohorts), insensitive attributes and labels. The choice of sensitive attribute(s) is presumably determined by human. We first define some terminologies used in this work. An **unfavorable** class in a sensitive attribute is a discriminated cohort. An **underrepresented** class in an insensitive attribute is the rarest among all classes. An instance is **underprivileged** if it is either unfavorable or underrepresented, or both. The aim of the weight assignment is to *give higher weights to positive instances if the instances are underprivileged*, and *give lower weights to positive instances if they are privileged*. The goal of RRW is to reduce discrimination by handling unfavorable attributes, while maintaining the overall prediction accuracy by caring about underrepresented ones. We start the discussion on weight assignment from problem formulation.

Let ${\left\{\left(X_{i},Y_{i},D_{i}\right)\right\}}_{i=1}^{n}$ be *n* samples from a joint distribution *p*_***X***,*Y*,*D*_ with domain X×Y×D. ***X***, *Y* and *D* denote insensitive attributes, labels, and sensitive attributes such as race and gender, respectively. In this work we focus on discrete and finite domain D and binary labels Y={0,1}. We only present the derivation under a univariate and binary scenario $D=\{d_{1},d_{2}\}$, while the proposed framework is applicable to higher dimensional D. We now define statistical parity, the notion of our fairness goal.

**Definition 1** *A binary classifier* $\hat{Y}\in Y$ *satisfies statistical parity with respect to a sensitive attribute* D∈D *if* $\hat{Y}$ *is independent of D*:

$$\begin{array}{c} Pr\left(\hat{Y}=1|D=d\right)=Pr\left(\hat{Y}=1|D=d^{\prime }\right)\;\;\forall d,\;d^{\prime }\in D. \end{array}$$

To distinguish categorical insensitive attributes from numerical ones, we introduce the categorical domain $X^{\left(c\right)}$ and the numerical domain $X^{\left(n\right)}$, and we have ***X*** = (***X***^(*c*)^, ***X***^(*n*)^) in $\left(X^{\left(c\right)},X^{\left(n\right)}\right)$. If both $X^{\left(c\right)}$ and $X^{\left(n\right)}$ are non-empty, then the weight assignment is a two-phase optimization problem: Phase I assigns weights under $X^{\left(c\right)}\times Y\times D$, and Phase II assigns weights under $X^{\left(n\right)}\times X^{\left(c\right)}\times Y\times D$. If only one type of ***X*** is observed, then we implement its corresponding phase alone.

### 3.1 Phase I optimization

Sample size plays a pivotal role in Phase I. Let *n*_***x***,*y*,*d*_ be the number of instances containing the triplet (***x***, *y*, *d*), and *n*_*y*,*d*_ be the number of instances containing the tuple (*y*, *d*). *n*_*y*_ and *n*_*d*_ are defined in a similar manner. Let $W_{1}:X^{\left(c\right)}\times Y\times D\to R^{+}$ be a weight function. We now assign a non-negative weight *W*_1_(***x***, *y*, *d*) to every instance $\left(x,y,d\right)\in X^{\left(c\right)}\times Y\times D$, so in total there are at most $|X^{\left(c\right)}\times Y\times D|$ weights to be sought. We impose the following constraints when we search for the optimal weights.

$$\begin{array}{c} \begin{array}{cc} & \sum_{\left(x,y,d\right)\in X^{\left(c\right)}\times Y\times D}W_{1}\left(x,y,d\right)n_{x,y,d}=n,\\ & \sum_{\left(x,d\right)\in X^{\left(c\right)}\times D}W_{1}\left(x,y,d\right)n_{x,y,d}=n_{y}\;\;\forall y\in Y,\\ & \sum_{\left(x,y\right)\in X^{\left(c\right)}\times Y}W_{1}\left(x,y,d\right)n_{x,y,d}=n_{d}\;\;\forall d\in D,\\ & W_{1}\left(x,y,d\right)\geq 0\;\;\forall \left(x,y,d\right)\in X^{\left(c\right)}\times Y\times D. \end{array} \end{array}$$ (1)

The weights are considered optimized if they have the following three properties.

**I. Independence guarantee.** To achieve this goal, we borrow strength from RW that attaches |Y×D| weights to all instances according to *Y* and *D*. Let *W*_1_(*y*, *d*) be the weights for all tuples (y,d)∈Y×D. RW can be summarized as

$$\begin{array}{c} W_{1}\left(y,d\right)=\frac{p_{Y}\left(y\right)p_{D}\left(d\right)}{p_{Y,D}\left(y,d\right)}=\frac{\left(n_{y}/n\right)\left(n_{d}/n\right)}{\left(n_{y,d}/n\right)}=\frac{n_{y}n_{d}}{n_{y,d}n}\;\;\forall \;\left(y,d\right)\in Y\times D. \end{array}$$ (2)

*W*_1_(*y*, *d*) in RW and *W*_1_(***x***, *y*, *d*) in RRW are closely related, as *W*_1_(*y*, *d*) can be regarded as a weighted average of *W*_1_(***x***, *y*, *d*) over all ***x***’s:

$$\begin{array}{c} W_{1}\left(y,d\right)=\sum_{x\in X^{\left(c\right)}}W_{1}\left(x,y,d\right)\frac{n_{x,y,d}}{n_{y,d}}\;\;\forall \;\left(y,d\right)\in Y\times D. \end{array}$$ (3)

If we combine Eqs (2) and (3), we obtain another equation that is intrinsically aligned with the first three equations in constraint set (1):

$$\begin{array}{c} \sum_{x\in X^{\left(c\right)}}W_{1}\left(x,y,d\right)n_{x,y,d}=\frac{n_{y}n_{d}}{n}\;\;\forall \;\left(y,d\right)\in Y\times D. \end{array}$$ (4)

The agreement between RW and *W*_1_(***x***, *y*, *d*) can be acknowledged in two ways. First, constraint set (1) about *W*_1_(***x***, *y*, *d*) are compatible with analysis on *W*_1_(*y*, *d*). Second, *Y* and *D* are independent. This can be visualized from the equivalence of the original distribution of *Y* and the weighted distribution of *Y* conditional on *D* by dividing both sides of Eq (4) by *n*_*d*_.

$$\begin{array}{c} p_{Y|D}^{w}\left(y|d\right)=\frac{\sum_{x\in X^{\left(c\right)}}W_{1}\left(x,y,d\right)n_{x,y,d}}{n_{d}}=\frac{n_{y}}{n}=p_{Y}\left(y\right). \end{array}$$

**II. Discrimination control.** The above conditional probability argument leads to discussion on the second goal. Group fairness is enforced when the difference between *p*_*Y*|*D*_(*y*|*d*_1_) and *p*_*Y*|*D*_(*y*|*d*_2_) is under control. If we extend our scope from *p*_*Y*|*D*_ to *p*_*Y*|***X***^(*c*)^, *D*_, we may further reduce the discrepancy by considering the relation between ***X***^(*c*)^ and *D*. We now introduce a weighted version of the conditional probability of *Y* given ***X***^*c*^ and *D*:

$$\begin{array}{c} p_{Y|X^{\left(c\right)},D}^{w}\left(y|x,d\right)=\frac{p_{X^{\left(c\right)},Y,D}^{w}\left(x,y,d\right)}{p_{X^{\left(c\right)},D}\left(x,d\right)}=\frac{W_{1}\left(x,y,d\right)n_{x,y,d}}{n_{x,d}}. \end{array}$$

Thus, we reduce the discrepancy of $p_{Y|D}^{w}$ between *d*_1_ and *d*_2_ for all (***x***, *y*) in $X^{\left(c\right)}\times Y$ by minimizing

$$\begin{array}{c} \sum_{\left(x,y\right)\in X^{\left(c\right)}\times Y}|\frac{W_{1}\left(x,y,d_{1}\right)n_{x,y,d_{1}}}{n_{x,d_{1}}}-\frac{W_{1}\left(x,y,d_{2}\right)n_{x,y,d_{2}}}{n_{x,d_{2}}}|\frac{n_{x}}{n}. \end{array}$$ (5)

**III. RW-Accuracy preservation.** To embrace the advantages of RW, we keep our optimal weights from being too deviated from RW’s solution by controlling the difference between *W*_1_(***x***, *y*, *d*) and $W_{1}\left(y,d\right)=\frac{n_{y}n_{d}}{n_{y,d}n}$ for all $\left(x,y,d\right)\in X^{\left(c\right)}\times Y\times D$:

$$\begin{array}{c} \sum_{\left(x,y,d\right)\in X^{\left(c\right)}\times Y\times D}|W_{1}\left(x,y,d\right)-\frac{n_{y}n_{d}}{n_{y,d}n}|. \end{array}$$ (6)

#### Optimization formulation

Putting constraint set (1), Eqs (4)–(6) together, we arrive at the optimization problem below for determining *W*_1_(***x***, *y*, *d*) for all $\left(x,y,d\right)\in X^{\left(c\right)}\times Y\times D$:

$$\begin{array}{cc} \text{min} & \sum_{\left(x,y\right)\in X^{\left(c\right)}\times Y}|\frac{W_{1}\left(x,y,d_{1}\right)n_{x,y,d_{1}}}{n_{x,d_{1}}}-\frac{W_{1}\left(x,y,d_{2}\right)n_{x,y,d_{2}}}{n_{x,d_{2}}}|\frac{n_{x}}{n}\\ & +\lambda \sum_{\left(x,y,d\right)\in X^{\left(c\right)}\times Y\times D}|W_{1}\left(x,y,d\right)-\frac{n_{y}n_{d}}{n_{y,d}n}|\\ \text{s.t.}\; & \;\;\sum_{x\in X^{\left(c\right)}}W_{1}\left(x,y,d\right)n_{x,y,d}=\frac{n_{y}n_{d}}{n}\;\;\forall \;\left(y,d\right)\in Y\times D,\\ & \;\;W_{1}\left(x,y,d\right)\geq 0\;\;\forall \;\left(x,y,d\right)\in X^{\left(c\right)}\times Y\times D, \end{array}$$ (7)

where λ > 0 is a tuning parameter. A smaller λ pulls the weights more toward group fairness, as less emphasis is put on maintaining the accuracy provided by RW. Indeed, RW is a special case of RRW.

**Proposition 1** *RRW is a generalized version of RW*.

**Proof 1** *If the* λ *in problem* (7) *is sufficiently large, the first summation in the objective function is dominated by the second summation. As the second summation is non-negative, the minimum value of the objective function is driven down to* 0. *As* λ → ∞,

$$\begin{array}{c} W_{1}\left(x,y,d\right)\to W_{1}\left(y,d\right)=\frac{n_{y}n_{d}}{n_{y,d}n} \end{array}$$

*for all* $\left(x,y,d\right)\in X^{\left(c\right)}\times Y\times D$. *Note that*

$$\begin{array}{c} \sum_{x\in X^{\left(c\right)}}W_{1}\left(x,y,d\right)n_{x,y,d}=\sum_{x\in X^{\left(c\right)}}\frac{n_{y}n_{d}n_{x,y,d}}{n_{y,d}n}=(\sum_{x\in X^{\left(c\right)}}n_{x,y,d})\frac{n_{y}n_{d}}{n_{y,d}n}=\frac{n_{y}n_{d}}{n}, \end{array}$$

*and* $\frac{n_{y}n_{d}}{n_{y,d}n}\geq 0$ *for all* (y,d)∈Y×D, *the optimal solution in RW satisfies all constraints provided by RRW. When* λ *is small, the optimal solution in RRW deviates from the one in RW*.

This optimization problem can be formulated to and therefore efficiently solved by linear programming. Here we address two practical concerns for implementation.

**What happen if not all combinations of *X*^(*c*)^, *Y* and *D* exist?** The underlying assumption of RRW is that *p*_***X***^(*c*)^, *Y*, *D*_ is known along with its marginals and conditionals. If there exists at least an (***x***, *d*) pair such that *n*_***x***, *d*_ = 0, the corresponding probability is undefined. To overcome this limitation, we exclude unobserved pairs in problem (7), and their weights will be 1, the original value without any treatment.

**What happen if there are too many *x*’s in $X^{\left(c\right)}$?** The time complexity of linear programming in problem (7) is exponential to the number of categorical attributes. To avoid the potential combinatorial explosion, we consider the bias corrected Cramér’s V [32], denoted by *V*, that measures pairwise association. For every $x_{j}\in X^{\left(c\right)}$, if ∑_*j*≠*k*_ *V*(*x*_*j*_, *x*_*k*_), the sum of its pairwise association with other attributes, is stronger than a certain threshold, then we claim the correlation between *x*_*j*_ and other attributes is so strong that *x*_*j*_ can either be excluded from both phases, or be viewed as numerical and handled by Phase II. This selection process ensures that the number of attributes handled by Phase I optimization remains sufficiently small for linear programming in high-dimensional categorical attribute spaces. In Section 4.4, we argue that the second route is preferred.

### 3.2 Phase II optimization

**Fig 1:**
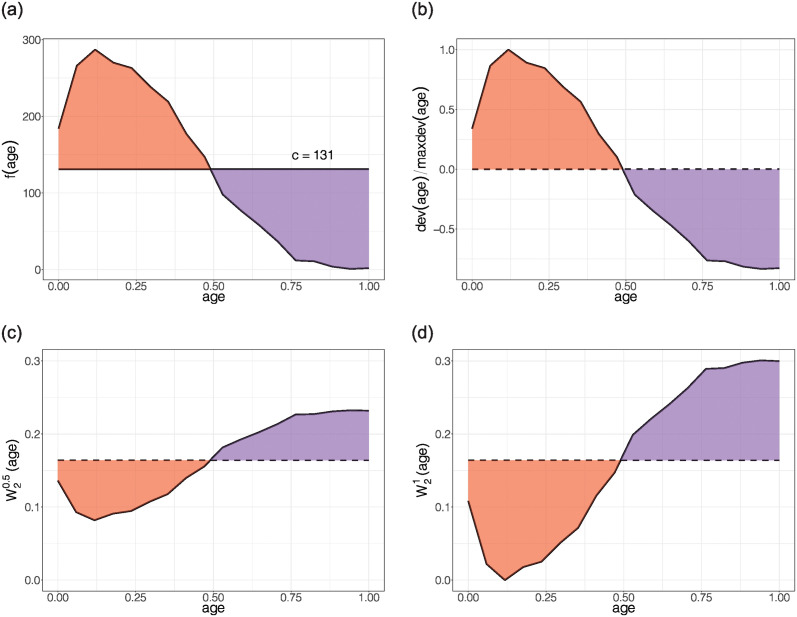
Graphical illustrations for the role of *c* with the Adult income data. Top-left: *f*(age) against age; top-right: dev(age)/max dev(age) against age; bottom-left: $W_{2}^{0.5}$(age) against age; and bottom-right: $W_{2}^{1}$(age) against age.

The probability distribution of numerical insensitive attributes plays an essential role in Phase II. Suppose *x*′ is a numerical insensitive attribute re-scaled to [0, 1]. For every $\left(x,y,d\right)\in X^{\left(c\right)}\times Y\times D$, let *f*(*x*′|***x***, *y*, *d*) be the frequency of $x^{\prime }\in X^{\left(n\right)}$ over all instances with (***x***, *y*, *d*). For simplicity, we write *f*(*x*′) = *f*(*x*′|***x***, *y*, *d*). When *x*′ is discrete, we obtain *f*(*x*′) by counting the frequencies of all available values in the training set. When *x*′ is continuous, we bucketize the values into equal-sized buckets, treating these buckets as discrete values as in the first scenario. The bucket size is determined by making each bucket as granular as possible while remaining non-empty. Given an unknown value *c* ranges from min *f*(*x*′) to max *f*(*x*′), we define dev_*c*_(*x*′) = *f*(*x*′) − *c* that measures the deviation of *f*(*x*′) from *c*, where *c* is determined by $\int_{0}^{1}{\text{dev}}_{c}\left(x^{\prime }\right)dx^{\prime }=0$. This can be solved by binary search over [min *f*(*x*′), max *f*(*x*′)]. Geometrically, *c* is a horizontal line across *f*(*x*′) whose positive and negative vertical distances to *f*(*x*′) integrate to 0. We provide a graphical illustration on the UCI Adult income data in the top-left plot of Fig 1 to demonstrate that *c* = 131 equalizes the red and purple areas.

As *c* is now fixed, we denote dev_*c*_(*x*′) by dev(*x*′) for simplicity. Next, let *t* ∈ [0, 1] be another unknown value to be solved. Let $W_{2}^{t}:X^{\left(n\right)}\times X^{\left(c\right)}\times Y\times D\to R^{+}$ be a weight function such that

$$\begin{array}{c} W_{2}^{t}\left(x^{\prime },x,y,d\right)=W_{1}\left(x,y,d\right)(1-t\frac{\text{dev}(x^{\prime })}{\text{max}\;\text{dev}(x^{\prime })}). \end{array}$$

For illustrative purposes, we express $W_{2}^{t}$ as a continuous function in this section. This function is not necessarily smooth in application as the weight is only defined for the points observed in the training set. When *t* = 1, $W_{2}^{1}$ is identical to the horizontal reflection of *f*(*x*′). When *t* ∈ [0, 1), geometrically, $W_{2}^{t}$ shrinks $W_{2}^{1}$ to *W*_1_ vertically by a factor of *t*. The last three plots in Fig 1 illustrate that $W_{2}^{t}\left(x^{\prime },x,y,d\right)$ and dev(*x*′)/max dev(*x*′) move in the opposite vertical direction. A nice property of $W_{2}^{t}$ is that for all *t* ∈ [0, 1],

$$\begin{array}{c} \int_{0}^{1}W_{2}^{t}\left(x^{\prime },x,y,d\right)dx^{\prime }=W_{1}\left(x,y,d\right). \end{array}$$

**Fig 2:**
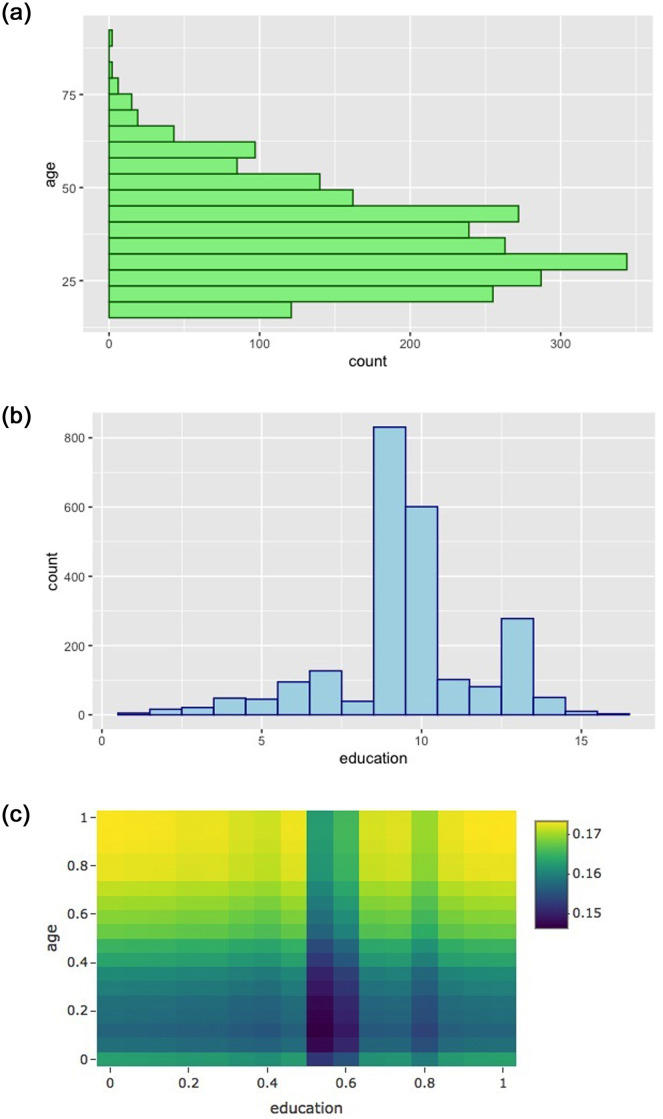
Top-left: *f*(age) for (non-white, female, < 50K income) individuals; top-right: *f*(education) for (non-white, female, < 50K income) individuals; and bottom: *W*_2_(non-white, female, < 50K, age, education) in the Adult data.

As shown in Fig 2, in the Adult data, lower $W_{2}^{t}$’s are assigned to more frequent combinations of age and education. The weighted average of $W_{2}^{t}$(race, gender, income, age, education) over age and education is exactly *W*_1_(race, gender, income).

Similar to expression (5), the optimized *t* minimizes the discrepancy of weighted conditional probability between *d*_1_ and *d*_2_:

$$\begin{array}{c} \begin{array}{cc} \sum_{\left(x,y\right)\in X^{\left(c\right)}\times Y} & \int_{0}^{1}|W_{2}^{t}\left(x^{\prime },x,y,d_{1}\right)p_{Y|X^{c},D}\left(y|x,d_{1}\right)\\ & -W_{2}^{t}\left(x^{\prime },x,y,d_{2}\right)p_{Y|X^{c},D}\left(y|x,d_{2}\right)|\frac{n_{x}}{n}dx^{\prime }. \end{array} \end{array}$$ (8)

This *t* can also be solved by binary search over [0, 1]. After we get the optimized value, we denote $W_{2}^{t}$ by *W*_2_ for simplicity.

In general, given *m* numerical insensitive attributes $x_{k}^{\prime }$, *k* = 1, …, *m*, we assume they have equal contribution to label prediction, as the classification model is not available under the pre-processing setting. The weight for $\left(x_{1}^{\prime },\ldots ,x_{m}^{\prime },x,y,d\right)$ is therefore

$$\begin{array}{c} W_{2}\left(\left(x_{1}^{\prime },\ldots ,x_{m}^{\prime },x,y,d\right)\right)=\frac{1}{m}\sum_{k=1}^{m}W_{2}\left(x_{k}^{\prime },x,y,d\right). \end{array}$$

It is easy to check that *W*_1_(***x***, *y*, *d*) is equivalent to

$$\begin{array}{c} \int_{0}^{1}\ldots \int_{0}^{1}W_{2}\left(\left(x_{1}^{\prime },\ldots ,x_{m}^{\prime },x,y,d\right)\right)dx_{m}^{\prime }\ldots dx_{1}^{\prime }. \end{array}$$

As every $W_{2}\left(\left(x_{k}^{\prime },x,y,d\right)\right)$ is independently solved in problem (8) for all $x_{k}^{\prime }$ and all $\left(x,y,d\right)\in X^{\left(c\right)}\times Y\times D$, and computation of $W_{2}\left(\left(x_{1}^{\prime },\ldots ,x_{m}^{\prime },x,y,d\right)\right)$ is $O\left(m\right)=O(|X^{\left(n\right)}|)$, the time complexity of Phase II optimization is $O(|X^{\left(n\right)}\times X^{\left(c\right)}\times Y\times D|)$. In high-dimensional categorical attribute spaces, where not all categorical attributes are handled by Phase I, Phase II has a linear time complexity relative to the number of remaining categorical attributes.

### 3.3 Training and prediction

RRW aims to help classifiers satisfy statistical parity by seeking independence between $\hat{y}$ and *d*. While the relationship between the observed *y* in the training set and *d* is established in the two optimization phases, minimizing the gap between *p*_*Y*|*D*_(*y*|*d*) and *p*_*Y*|*D*_(*y*|*d*′) for all *d* and *d*′, we also need to establish a connection between the observed *y* in the training set and the predicted $\hat{y}$ in the test set. This connection requires the assumption that the training and test sets share the same conditional distribution of labels given the sensitive attribute. In other words, statistical parity can be achieved when

$$\begin{array}{c} p_{\hat{Y}|D}\left(\hat{y}|d\right)\approx p_{Y|D}\left(y|d\right)\;\forall d\in D. \end{array}$$

In practice, when we train a model with the assigned weights, we update the weights of training instances, and feed them to the classification model. To predict labels of test instances, we run the model on testing data as usual without any extra steps.

## 4 Experimental results

We evaluate the performance of RRW on both synthetic and benchmark datasets, and conduct an extensive comparison among two baselines, six pre-processing approaches and one in-processing method on three fairness metrics. Denote predicted labels by $\tilde{Y}$. The first fairness measure is motivated by the “80% rule” [33] and statistical parity [15–17]:

$$\begin{array}{c} {\text{Discrimination}}_{\text{sp}}=\underset{d,d^{\prime }\in D}{\text{max}}\;|\frac{p_{\tilde{Y}|D}\left(1|d\right)}{p_{\tilde{Y}|D}\left(1|d^{\prime }\right)}-1|. \end{array}$$ (9)

The second measure is disparate impact [30, 31]:

$$\begin{array}{c} {\text{Discrimination}}_{\text{di}}=\frac{p_{\tilde{Y}|D}\left(1|\text{unfavorable}\;d\right)}{p_{\tilde{Y}|D}\left(1|\text{favorable}\;d\right)}. \end{array}$$ (10)

It focuses on the proportions of two cohorts that receive the positive outcome. The last measure is the false discovery rate. We report the trade-off between the empirical discrimination on test set and the empirical accuracy, measured by the Area under ROC (AUC). As the accuracy versus discrimination pattern remains stable across various classifiers such as logistic regression, support vector machine, random forest and Gaussian naïve bayes for all pre-processing methods [6, 12], we only present results on logistic regression. All experiments are conducted on a machine with an Intel Xeon E5-2690 2.90GHz CPU and 256GB RAM.

### 4.1 Phase I simulation

#### Data

Let *D* = (*D*_1_, …, *D*_*n*_), *D*_*i*_ ∈ {+1, −1} for all *i* be a sensitive attribute vector of length *n*. 80% entries are set to be unfavorable. Categorical insensitive attributes {*X*_*ij*_}, *i* = 1, …, *n*, *j* = 1, …, *q*−1 are randomly generated, and set to be binary with 1/2 chance for each outcome in {+1, −1}. *D* and all *X*’s are independent. Let *Y* = (*Y*_1_, …, *Y*_*n*_) be the true label vector of length *n*. We assign different coefficients, ${\left\{\beta_{j}\right\}}_{j=0}^{q-1},\beta_{0}=0.15$ and *β*_*j*_ = 0.01 × *j* for *j* = 1, …, *q*−1, to different outcomes of *D* and *X* so that the linear combination of *D*_*i*_ and *X*_*ij*_ can be used to determine the true labels by treating *Y*_*i*_ as a Bernoulli random variable

$$\begin{array}{c} Y_{i}∼\text{Bernoulli}\left(1/\left(1+\text{exp}\left(-\left(\beta_{0}D_{i}+\sum_{j=1}^{q-1}\beta_{j}X_{ij}\right)\right)\right)\right) \end{array}$$

for all *i*. We randomly split 80% and 20% of all instances into training and test sets.

#### Implementation

The parameters in LFR [12] are chosen according to its authors’ recommendation: *A*_*x*_ = 0.01, *A*_*y*_ and *A*_*z*_ ∈ {0.1, 0.5, 1, 5, 10}. The regularization parameter in DIR [13] is selected based on balanced error rate. The parameters in OPT [6] are chosen as its authors suggest whenever they are publicly available. To demonstrate the impact of sample size on computational time, we pick seven different values for *n* from 10^5^ to 10^7^, and set *q* = 5. We compare RRW with LFR, DIR and OPT and leave out RW, as sample size does not affect the speed of RW. We consider Eq (9) as the fairness measure.

#### Results

**Fig 3:**
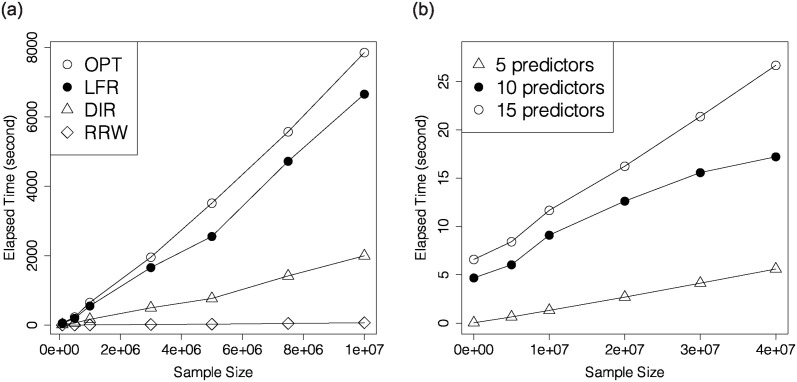
Elapsed time versus sample size for OPT, LFR, DIR and RRW when *q* = 5 (left), and elapsed time versus sample size for RRW when *q* = 5, 10, 15 (right).

The left plot in Fig 3 reveals that OPT and LFR consume more time than DIR and RRW. Even though there are $|X^{\left(c\right)}\times Y\times D|$ weights to be sought in RRW, the underlying data representations in OPT and LFR take even longer time than the reweighing in RRW. Little computational burden is added to the linear programming step in RRW, as all *n*_*x*,*y*,*d*_’s are summarized in the coefficient matrix before the optimization is conducted.

To investigate the influence of the number of attributes for RRW, we pick different values for *n* from 10^4^ to 4 × 10^7^, and *q* = 5, 10, 15. As we have mentioned in Section 3.1, we select the 10 most uncorrelated insensitive attributes out of 14 when *q* = 15. The right plot in Fig 3 illustrates that weights can be assigned to 40 million instances within 30 seconds. Although elapsed time grows linearly as sample size increases, the growth rate is indeed significantly lower than those of LFR, DIR and OPT, which is demonstrated by the left plot in Fig 3.

To compare their performance on fairness and accuracy, we set *q* = 5 (1 sensitive attribute and 4 categorical insensitive attributes), *n* = 10^4^, and set the AUC for all methods to be 0.505. We report Discrimination_sp_ under the best sets of parameters in Table 1. RRW outperforms all other methods. Furthermore, for *n* = 10^5^ and *q* = 5, 10, 15, as shown in Table 2, under the best λ, RRW outperforms RW in Discrimination_sp_ by at least 25%.

**Table 1:** Discrimination for *q* = 5 and *n* = 10^4^ under AUC = 0.505.

Method	RW	LFR	OPT	DIR	RRW
Discrimination	0.004	0.008	0.006	0.037	0.003

**Table 2:** AUC and discrimination for full, RW and RRW and *q* = 5, 10, 15.

*q*	Method	AUC	Discrimination_sp_
5	Full	0.528	0.166
5	RW	0.497	0.004
5	RRW	**0.505**	**0.003**
10	Full	0.513	0.201
10	RW	0.503	0.015
10	RRW	**0.504**	**0.007**
15	Full	0.519	0.238
15	RW	**0.507**	0.006
15	RRW	0.504	**0.003**

### 4.2 Phase II simulation

#### Data

Continue with the above setting. Let $\left\{X_{ik}^{\prime }\right\}$, *i* = 1, …, *n*, *k* = 1, …, *m* be independent and randomly generated numerical insensitive attributes, where $X_{ik}^{\prime }$ follows a Beta(*k*, *m* + 1 − *k*) distribution. We assign coefficients ${\left\{\gamma_{k}\right\}}_{k=1}^{m},\gamma_{k}=0.01\times k$ for *k* = 1, …, *m*, to $X_{ik}^{\prime }$, so the linear combination of *D*_*i*_, *X*_*ij*_ and $X_{ik}^{\prime }$ can be used to determine the true labels by treating *Y*_*i*_ as a Bernoulli random variable with parameter

$$\begin{array}{c} 1/(1+\text{exp}\left(-\left(\beta_{0}D_{i}+\sum_{j=1}^{q-1}\beta_{j}X_{ij}+\sum_{k=1}^{m}\gamma_{k}X_{ik}^{\prime }\right)\right)) \end{array}$$

for all *i*. We randomly split 80% and 20% of all instances into training and test sets.

#### Results

**Fig 4:**
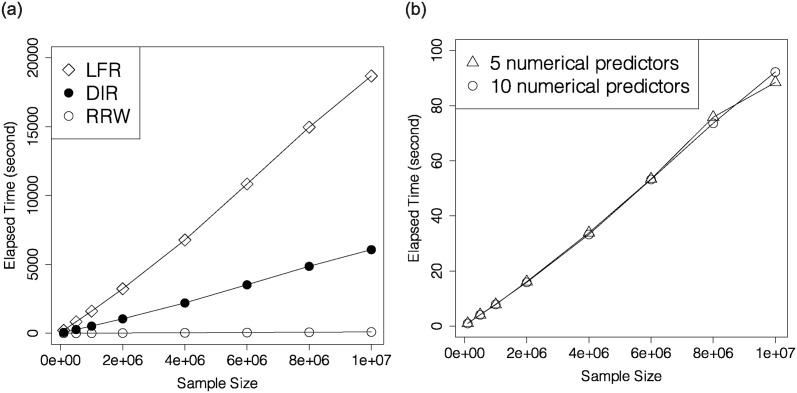
Elapsed time versus sample size for LFR, DIR and RRW when *q* = 5 and *m* = 10 (left), and elapsed time versus sample size for RRW when *q* = 5 and *m* = 5, 10 (right).

To demonstrate the impact of sample size on computational time in Phase II, we choose 7 different values for *n* arranging from 10^5^ to 10^7^, *q* = 5 and *m* = 10. We compare RRW with LFR and DIR, and leave out OPT, as OPT is not compatible with numerical attributes. The left plot in Fig 4 reveals that LFR and DIR take more time than RRW. To investigate the influence of the number of numerical attributes in Phase II, we pick different values for *n* from 10^5^ to 10^7^, *q* = 5 and *m* = 5, 10. The right plot in Fig 4 illustrates that under this setting, increment of *m* does not lead to increment of computational time. All weights can be assigned within 100 seconds.

**Fig 5:**
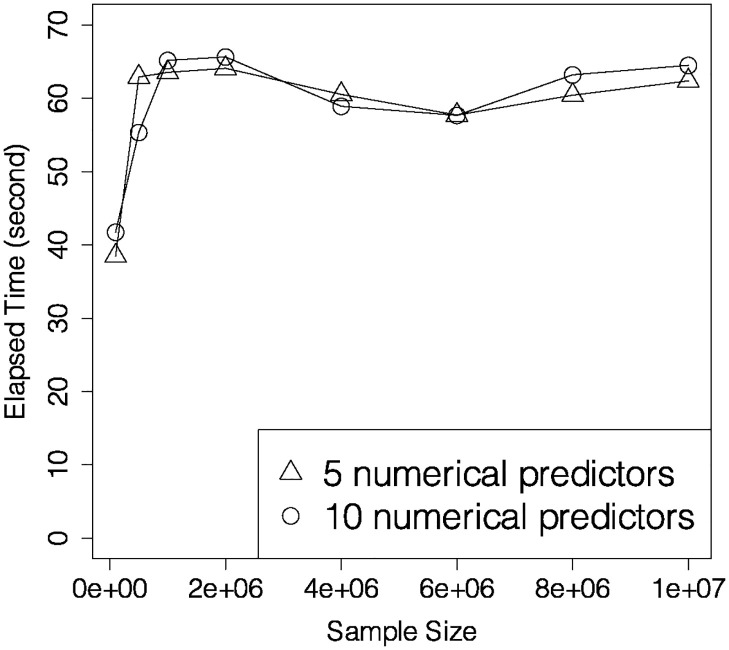
Ratio of time spent in Phase II over Phase I for RRW when *q* = 5 and *m* = 5, 10.

To better understand this observation, we look at the ratio of time spent in Phase II over Phase I. Fig 5 reveals that the ratio is around 64 when *n* is large. This ratio validates the theoretical time complexity of Phase I and Phase II optimizations, because $|X^{c}\times Y\times D|=64$ in our simulation setting, which is larger than *m*. Therefore, when the numbers of categorical and numerical attributes are both large, one can take some of the categorical attributes as numerical, and pass them to Phase II to reduce computational time.

To compare the performance RRW, LFR and DIRs on fairness and accuracy, we set *q* = 5, *m* = 10 and *n* = 10^5^, and set the AUC for all methods to be 0.505. We report Discrimination under the best sets of parameters in Table 3. Once again, RRW outperforms the other two methods. Tables 1 and 3 show that Phase I potentially plays the dominant role for bias mitigation.

**Table 3:** Discrimination for *q* = 5, *m* = 10 and *n* = 10^5^ under AUC = 0.505.

Method	LFR	DIR	RRW
Discrimination	0.007	0.011	**0.003**

### 4.3 Real data experiments on statistical parity

#### Data

Here we present the accuracy-fairness trade-off of two baselines and four pre-processing methods on three benchmark datasets: ProPublica’s COMPAS recidivism data, the UCI Adult income data, and the UCI German credit data. Since OPT focuses on categorical predictors, two numerical attributes in Adult are categorized by their authors. In our experiments, we work on both the original and the categorized versions so as to set our comparison on the same basis. Since DIR works better with numerical attributes than categorical ones, applying DIR on categorical attributes may hinder its performance.

#### Implementation

“Full” uses all attributes to train and test. “Drop” leaves out *race* in COMPAS, *gender* in Adult, and *age category* in German, which are considered sensitive attributes. RW calculates the weights without tuning or regularization. LFR selects *A*_*x*_ = 0.01 for group fairness, and we apply grid search for *A*_*y*_ (prediction accuracy) and *A*_*z*_ (individual fairness) from {0.1, 0.5, 1, 5, 10} to get its top outcomes in all three datasets. The regularization parameter in DIR is irresponsive when all attributes are categorical, so we only present one outcome in each case. The tuning parameter in RRW takes values from the range [0.49, 0.82] in COMPAS, [0.71, 0.90] in Adult, and [0.75, 0.95] in German, so that the trade-offs are clearly visualized in a range in all three cases. OPT requires a large amount of calibration. Since experiments in their paper are conducted in COMPAS and Adult, we adopt their choices of tuning parameters. In German, the distortion function is chosen as default in their program, and we select 0.2, 0.2 and 0 as the corresponding probability bounds *c*_*d*,*x*,*y*_. We use three levels of discrimination control, *ϵ* ∈ {0.01, 0.05, 0.1}. We consider Eq (9) as the fairness measure.

#### Results

**Fig 6:**
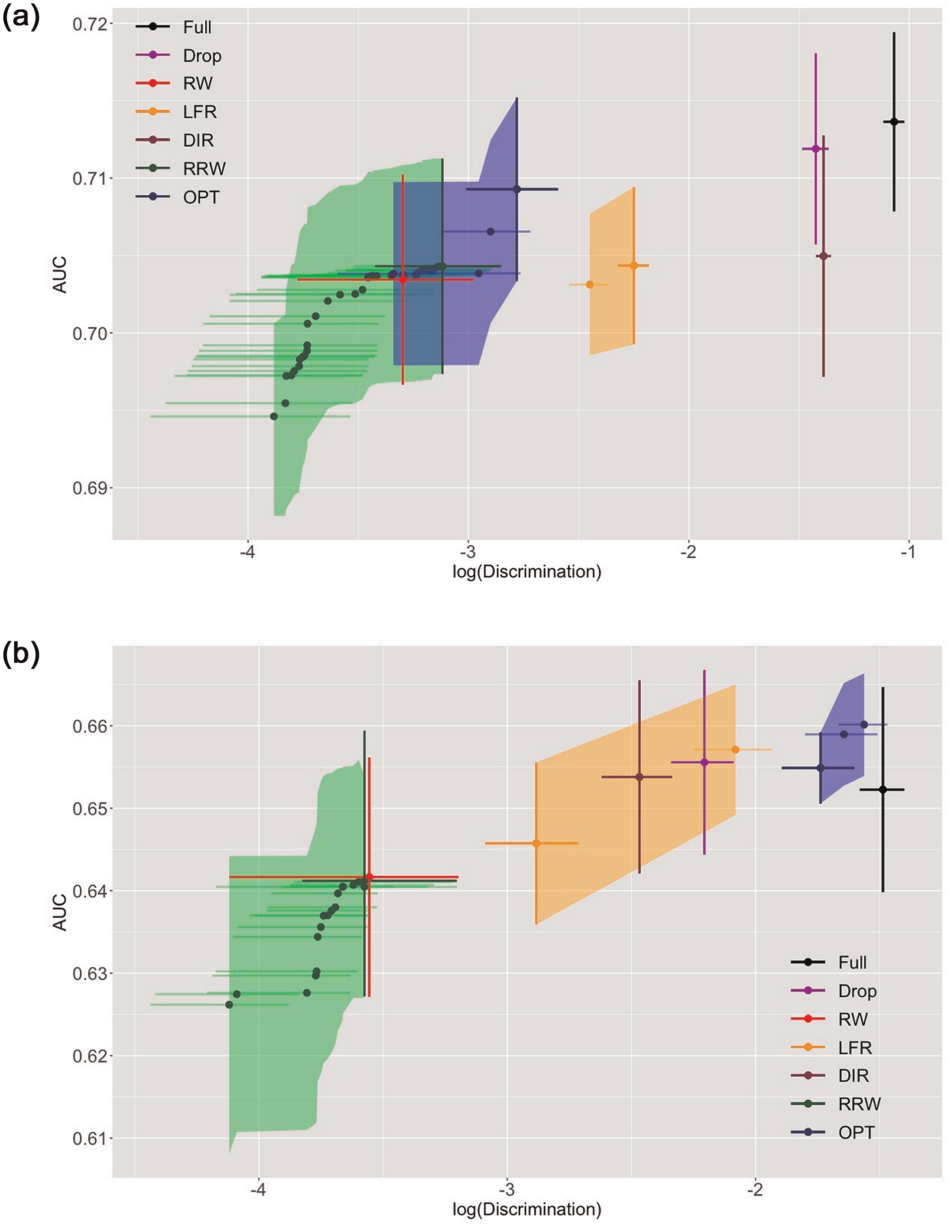
AUC versus Log(Discrimination) for COMPAS (top) and GERMAN (bottom).

**Fig 7:**
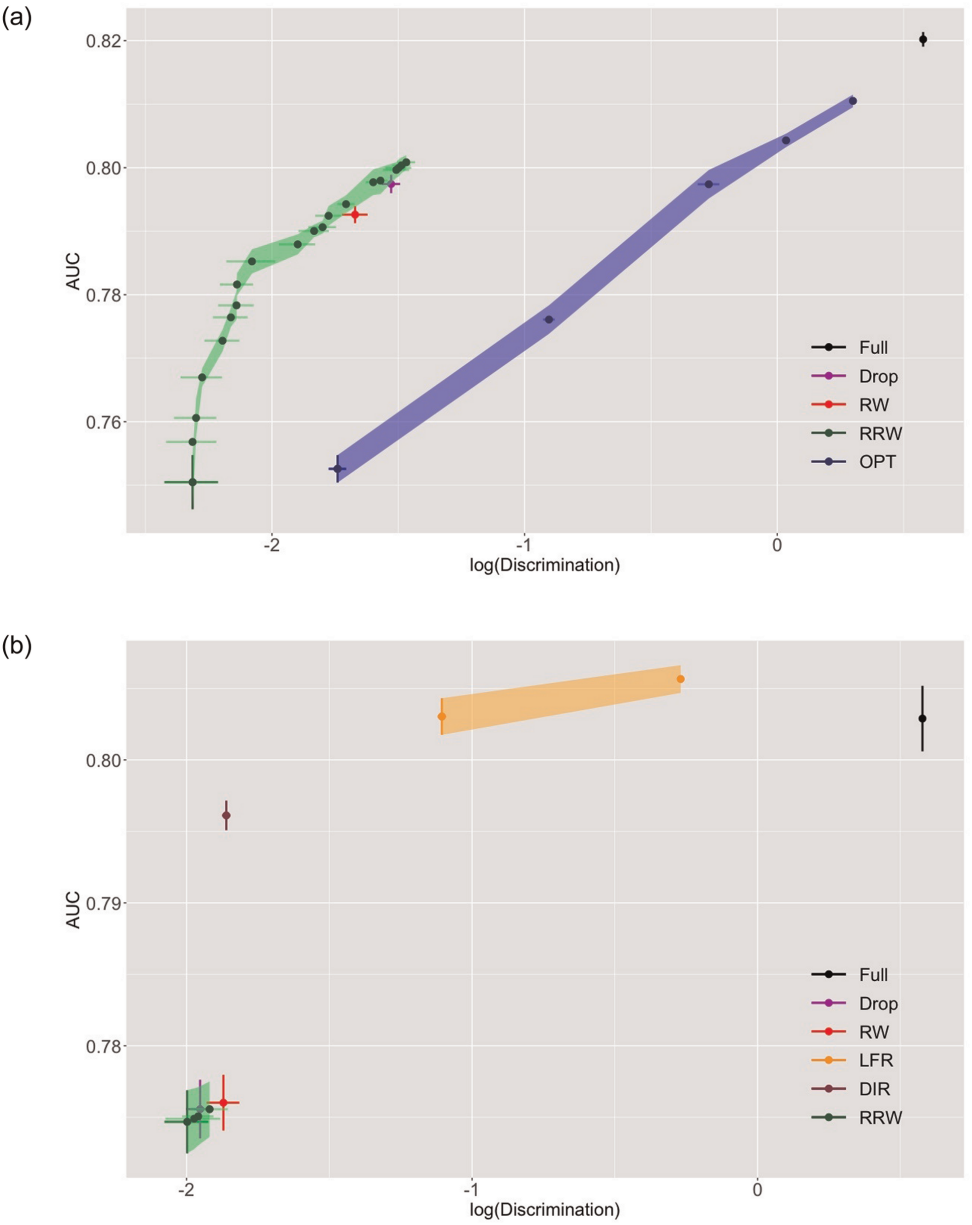
AUC versus Log(Discrimination) for categorized Adult (top) and original Adult (bottom).

We plot the experimental results in Figs 6 and 7. The average log-discrimination score, which is the natural log of Eq (9), and AUC are calculated by 5-fold cross validation. Error bars and vertical shades represent values that are one standard error deviated from their means. As λ increases, the trace in green representing RRW gradually moves from the lower left corner to the upper right.

In COMPAS presented by Fig 6 (top), “Drop” stays close to “Full”, which implies that simply ignoring the sensitive attribute does not help reduce discrimination in this dataset. DIR is close to the cluster of two baselines. LFR lies to the left of the cluster. RRW, RW and OPT are the top three performers in this case. They all have similar standard errors in both directions. RRW and OPT present the fairness-accuracy trade-off extensively, and RRW in general shows a greater discrimination reduction than OPT. Note that RRW marginally outperforms RW by reducing discrimination to a certain extent at the same accuracy level.

German has only 1000 samples, the least number of instances among the three datasets. It can be visualized from Fig 6 (bottom) that all methods have large standard errors. “Drop” and all other modification-based methods enjoy better accuracy and fairness than “Full”. “Drop”, DIR and OPT are within the same cluster on the top right corner. LFR and RRW demonstrate a wide range of layout, indicating that the trade-off is effectively monitored. Moreover, since the performance of RW and RRW does not rely on the richness of sample space, they outperform all other methods in bias mitigation. However, since the proportions of all available attribute-label combinations are unstable when different training and test sets splits are performed within a low number of training samples, and reweighing methods rely heavily on sample sizes, the one standard error bars of RW and RRW are wider than other methods. This observation indicates that the conditional distributions of labels given the sensitive attribute vary between the training and test set under 5-fold cross-validation.

Fig 7 (top) illustrates the performance on categorized Adult. “Drop” stays close to RW, and the curve of RRW is to their top left. The widespread ranges where RRW and OPT lie indicate that the trade-off is well monitored by their parameter setting, but the range of OPT is to the right of RRW. It is clear that RRW outperforms other methods in both fairness and accuracy. In original Adult illustrated in Fig 7 (bottom), DIR takes the upper left corner to the most extent compared to all other approaches, which shows that it is better at handling numerical attributes than RRW. While “Drop”, RW and RRW lie in the same region as in categorized Adult, RRW is marginally to the left of the other two. It is worth noting that LFR is in the middle of the RRW cluster and “Full” in terms of discrimination. Nevertheless, it has the highest level of accuracy among all algorithms.

These real data experiments demonstrate that RRW can confidently outperform modification methods in reducing discrimination when the majority of insensitive attributes are categorical and the training dataset is large enough to ensure that most attribute-label combinations are well represented. When most attributes are categorical, the intermediate representation [12], feature alteration [13], and probabilistic transformation [6] learned by modification methods are limited by the total number of attribute-label combinations. In contrast, RRW effectively increases the representativeness of underprivileged instances. Once the weights are correctly adjusted by RRW, the limited number of attribute-label combinations is not a disadvantage. However, when more attributes are numerical, attribute-label combinations become more granular, resulting in some combinations being so rare that the weights learned by RRW may overreact to non-zero small counts. Consequently, RRW reduces bias but incurs a larger reduction in accuracy compared to modification methods.

**How does the inclusion of insensitive attributes refine weight assignments?** Given that the novelty of RRW lies in using both sensitive and insensitive attributes to refine weights, we now examine COMPAS more closely to understand how these insensitive attributes contribute to up-weighting or down-weighting a sample.

COMPAS contains a total of 5,278 data points. Charge degree, prior counts, and age category are three categorical insensitive attributes (**X**). For charge degree, 3,440 samples are classified as felonies (F) and 1,838 as misdemeanors (M), making M the underrepresented class. Regarding prior counts, 1,667 samples have 0 counts (0), 1,953 samples have 1 to 3 counts (1–3), and 1,658 samples have more than three counts (> 3), making > 3 the underrepresented class. In terms of age category, 1,156 samples are younger than 25 (< 25), 3,026 are between 25 and 45 (25–45), and 1,096 are older than 45 (> 45), making the > 45 class underrepresented. Race is the sensitive attribute (D), with African-Americans (AA) considered the unfavorable class and Caucasians (C) the favorable class. Regarding labels (Y), positive samples refer to those without recidivism (0), while negative samples refer to those with recidivism (1).

Table 4 illustrates scenarios where a sample is either up-weighted or down-weighted. For instance, while the RW weight for (AA, 0) is 1.132, considering prior counts, RRW assigns a higher weight of 2.398 to (F, > 3, 25–45, AA, 0) compared to (F, 1–3, 25–45, AA, 0), as > 3 is more underrepresented than 1–3. Similarly, while the RW weight for (AA, 1) is 0.898, considering charge degree, RRW assigns a lower weight of 0.494 to (M, > 3, 25–45, AA, 1) than to (F, > 3, 25–45, AA, 1), as M is more underrepresented than F. The extent of weight refinement from RW to RRW depends on the tuning parameter λ. For example, the RRW weights for (F, 0, 25–45, C, 0) and (F, 1–3, > 45, C, 0) are identical when λ = 0.5, but a smaller λ could differentiate their RRW weights.

**Table 4:** RW and RRW weights for a subset of sample patterns in COMPAS when λ = 0.5.

(**X**, *D*, *Y*)	Characteristic	RW Weight	RRW Weight
(F, > 3, 25–45, AA, 0)	underprivileged and positive	1.132	2.398
(F, 1–3, 25–45, AA, 0)	underprivileged and positive	1.132	1.132
(M, > 3, 25–45, AA, 1)	underprivileged and negative	0.898	0.494
(F, > 3, 25–45, AA, 1)	underprivileged and negative	0.898	0.898
(F, 0, 25–45, C, 0)	privileged and positive	0.851	0.851
(F, 1–3, > 45, C, 0)	underprivileged and positive	0.851	0.851
(F, 0, 25–45, C, 1)	privileged and negative	1.207	1.819
(M, 0, 25–45, C, 1)	underprivileged and negative	1.207	1.207

### 4.4 Alleviate computational burden in Phase I optimization

When the number of categorical attributes grows, the efficiency of linear programming in Phase I is limited by its exponential time complexity. To alleviate this computational burden, we have two potential solutions that are related to the bias corrected Cramér’s V [32]. By ranking all categorical attributes starting from the one with the strongest correlation, we can either exclude the top attributes from both phases, or exclude them from Phase I and include them in Phase II. We consider Eq (9) as the fairness measure.

**Fig 8:**
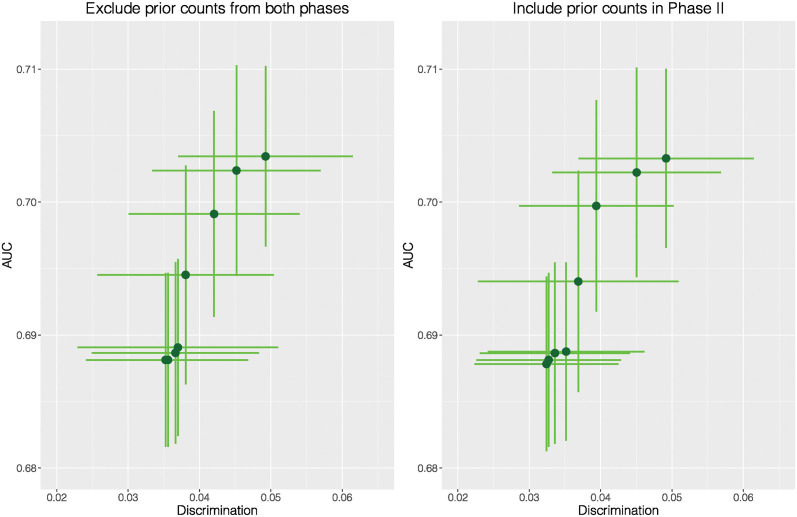
AUC versus Discrimination for COMPAS when prior counts is treated in two different ways.

In COMPAS, as prior counts, age and charge degree follow a descending order in correlation, we start with prior counts. Given a fixed set of tuning parameter λ’s, if we simply exclude prior counts in Phases I and II, the spans of mean AUC and the mean Discrimination are 0.015 and 0.014, respectively. If we treat prior counts in Phase II, the spans of both metrics are 0.015 and 0.017. While performance in accuracy looks similar, the second approach provides more room for improving fairness. The subtle difference is visualized in Fig 8.

**Fig 9:**
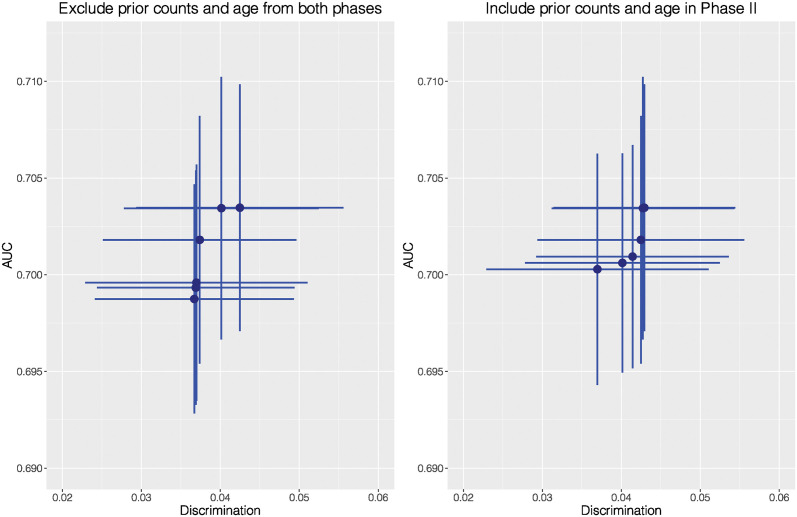
AUC versus Discrimination for COMPAS when prior counts and age are treated in two different ways.

If we ignore both prior counts and age in both phases, the span of the mean Discrimination is 0.006, and the mean AUC can be lower than 0.70. By comparing Figs 8 and 9 with the left plot of Fig 6, we can see the spans of the mean AUC and Discrimination get narrower as more categorical attributes are excluded from Phase I. Nevertheless, if we move both attributes to Phase II, the Discrimination span is 0.006, and the mean AUC is higher than 0.70. The difference is demonstrated in Fig 9.

If we ignore all three categorical insensitive attributes in both phases, then only race and recidivism are considered in Phase I. Consequently, the weights generated in Phase I will be identical to those produced by RW. Thus, λ cannot monitor the trade-off between fairness and accuracy.

In a word, to alleviate the computational burden brought up by the Phase I optimization at the cost of restricted fairness-accuracy trade-off, it is more desirable to view the categorical insensitive attributes with strong correlation as numerical and pass them to Phase II than to ignore them in both phases. This analysis also confirms that Phase I plays the dominant role over Phase II for both bias mitigation and accuracy reservation.

### 4.5 Additional experiments on disparate impact

To check if RRW is robust to other fairness metrics, we compare it with the other three post-processing techniques on disparate impact in Eq (10).

- Equalized Odds Post-processing (EOP) [19] optimizes equalized odds by changing output labels. It randomly flips prediction of test instances under the constraint that the privileged and underprivileged cohorts have the same false negative rate and the same false positive rate.
- Reject Option Classification (ROC) [34] gives positive outcomes to underprivileged cohorts and negative outcomes to privileged cohorts in a confidence band around the decision boundary with the highest uncertainty.
- Individual and Group Debiasing post-processing (IGD) [22] uses an individual bias detector to prioritize instances to improve disparate impact.

**Fig 10:**
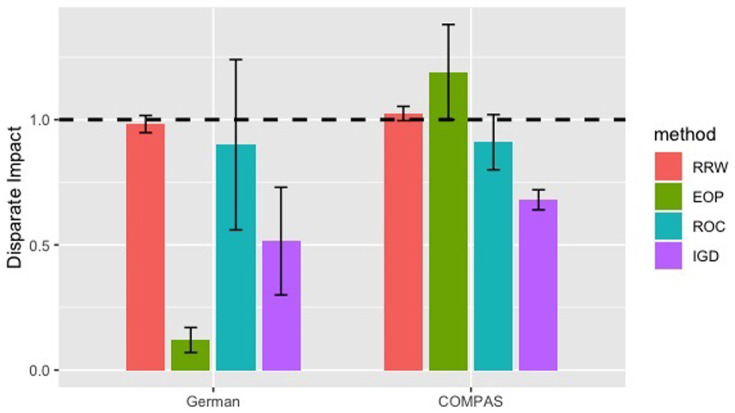
Disparate impact of RRW, EOP, ROC and IGD. Colored bars represent the means, vertical error bars the range within 1 standard deviation, and dashed line the best possible disparate impact 1.

**Fig 11:**
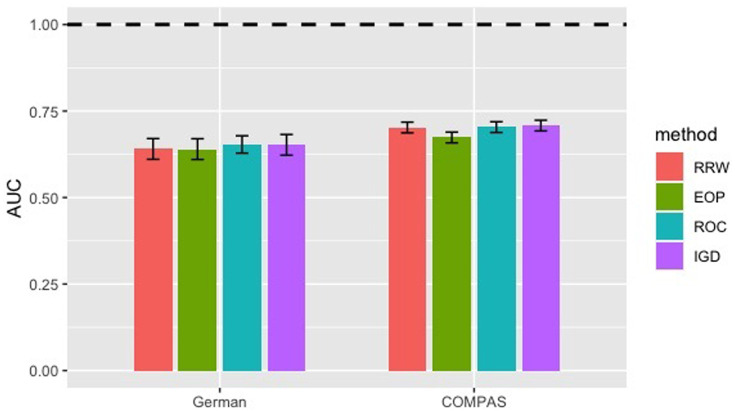
AUC of RRW, EOP, ROC and IGD. Colored bars represent the means, vertical error bars the range within 1 standard deviation, and dashed line the best possible AUC 1.

We optimize the parameters in all four methods to deliver a fair comparison. As shown in Figs 10 and 11, RRW improves the most fairness with the smallest standard deviation while maintaining accuracy in German and Adult. ROC is the second best in improving disparate impact. IGD is the best in preserving AUC. EOP performs poorly on disparate impact in German, because it optimizes equalized odds that does not necessarily give ideal disparate impact.

### 4.6 Comparison with an in-processing technique

The meta fair classifier [20] is an in-processing technique that takes the fairness metric as part of the input and returns a classifier optimized with respect to that fairness metric. To deliver the best outcome when we compare it with RRW in terms of false discovery rate, we use *τ*_fdr_ defined in [20] as the input, and return *γ*_fdr_ as the outcome, which is the ratio of false discovery rate of different sensitive attributes. We use the original train-test split of the categorized Adult data. For the meta fair Algo 1-FDR, the best accuracy-*γ*_fdr_ is 0.75-0.93, whereas the best outcome for RRW is 0.75-0.88. This reveals that in-processing techniques may still have better performance than pre-processing methods.

## 5 Conclusions

We propose a straightforward data pre-processing technique named Refined Reweighing that assigns customized weights to training instances in order to reduce discrimination against unfavorable groups. Simulation studies on large-scale synthetic data show that our method is more scalable than some other approaches in the literature. The extensive exploration on three real datasets indicates that our method is capable of controlling trade-off between fairness and accuracy. RRW can outperform modification methods in reducing discrimination when the training dataset is large enough to ensure that most attribute-label combinations are well represented and the majority of insensitive attributes are categorical. Furthermore, the single tuning parameter in Phase I optimization helps model users easily control the amount of cost in accuracy they would pay for fairness in a timely manner under the categorical setting. Phase II optimization extends applicability to numerical attributes. We are confident that the feasibility of our approach allows efficient and practical application on real-world problems in various domains.

Despite the promising experimental results, we acknowledge that statistical parity has become less popular due to the inherent tension between fairness and accuracy. For other group fairness notions such as equal opportunity, the probability being equated across cohorts is of a “correct” label rather than a “particular” label. Investigating the extensions to other group fairness notions and further reducing the cost in accuracy in pursuing fairness are desirable for future work.
